# Ultrasound-guided kidney biopsy: a review of what operators need to know

**DOI:** 10.3389/fneph.2026.1861024

**Published:** 2026-08-24

**Authors:** Husain Alturkistani

**Affiliations:** Radiology and Medical Imaging Department, College of Medicine, King Saud University, Riyadh, Saudi Arabia

**Keywords:** acute kidney injury, chronic kidney disease, kidney biopsy, percutaneous renal biopsy, ultrasound guidance

## Abstract

Ultrasound-guided kidney biopsy is a key procedure for diagnosing renal parenchymal disease and assessing renal involvement in systemic disorders. This review summarizes indications and contraindications, patient preparation, procedural technique, and post-biopsy monitoring, with emphasis on complication prevention and risk mitigation. Pre-biopsy ultrasound is essential to assess renal size, morphology, and anatomic variants and to plan a safe needle trajectory. Biopsy is typically performed from the lower pole renal cortex in the prone position using a spring-loaded core needle after local anesthesia. Bleeding is the principal complication, so structured observation after the procedure is mandatory.

## Introduction

The development and widespread application of interventional radiology techniques have made them vital for diagnosing and treating numerous disorders. In 1901, the first surgical renal biopsy was performed for the treatment of Bright disease (1). Recently, improvements and new techniques have been developed, including open surgical, laparoscopic, transjugular, transurethral, and percutaneous procedures. Percutaneous renal biopsy (PRB) is currently the preferred technique. However, other approaches should be considered when the percutaneous approach fails or is contraindicated (2).

Since the first procedure by Iversen and Brun in 1951, which was performed under fluoroscopic guidance using antegrade pyelography to obtain parenchymal samples, with a reported success rate of approximately 40%, percutaneous kidney biopsy has contributed to the development of diagnosis, prognosis, and management of patients with kidney disorders for decades (3, 4).

However, despite advancements in technology, this interventional procedure still carries several risks. Owing to the high vascularity of the kidney and its deep retroperitoneal position, which makes it difficult to compress the biopsy site, bleeding complications remain common. Hypertension, inflammatory status, and platelet dysfunction linked to uremia are common in patients with renal disorders, all of which increase the risk of bleeding (5–7).

### Indications for kidney biopsy

- Urologically unexplained macroscopic hematuria.
- Proteinuria.
- Nephrotic syndrome.
- Impaired renal function tests.
- Systemic diseases with potential renal involvement
  - Hypertension, Diabetes Mellitus.
  - Multiple myeloma.
  - Autoimmune diseases (systemic lupus erythematosus (SLE), antiphospholipid syndrome, scleroderma) (**Table 1**) (8).

**Table 1:** Indications, rationale, and contraindications of kidney biopsy.

Indications for kidney biopsy
Urologically unexplained macroscopic hematuria
Proteinuria
Nephrotic syndrome
Impaired renal function tests
Systemic diseases with potential renal involvement
Hypertension
Diabetes mellitus
Multiple myeloma
Autoimmune diseases, including systemic lupus erythematosus, antiphospholipid syndrome, and scleroderma
Rationale for performing kidney biopsy
Diagnosis or treatment guidance can be provided mainly through biopsy
Suspected diseases have a history of significant morbidity and/or mortality
Disease can be improved by therapy
Biopsy can result in a different diagnosis that requires different treatment
Treatments have acceptable adverse-event profiles
The risks associated with the procedure are acceptable
Contraindications of kidney biopsy
Renal atrophy
End-stage renal disease
Bilateral multiple renal cysts
Uncorrectable coagulopathy or thrombocytopenia
Patients on anticoagulant or antiplatelet aggregation therapy
Severe uncontrolled hypertension
Upper urinary tract infection
Uncooperative patient
Obstructive nephropathy with hydronephrosis
Solitary native kidney
Cystic or structurally abnormal kidney

### Kidney biopsy is an invasive diagnostic procedure that should only be recommended if

- Diagnosis or treatment guidance can be provided mainly through biopsy.
- Suspected diseases have a history of significant morbidity and/or mortality.
- Disease can be improved by therapy.
- Biopsy can result in a different diagnosis that requires different treatment.
- Treatments have acceptable adverse event profiles.
- The risks associated with the procedure are acceptable.

In isolated microscopic hematuria or low-grade proteinuria cases (< 0.5-1.0 g/day), renal biopsy is often not recommended unless there is another indication, such as deranged renal function tests (9).

### Contraindications of kidney biopsy

- Renal atrophy.
- End-stage renal disease.
- Bilateral multiple renal cysts.
- Uncorrectable coagulopathy or thrombocytopenia.
- Patients on anticoagulant or antiplatelet aggregation therapy.
- Severe uncontrolled hypertension.
- Upper urinary tract infection (**Table 1**).
- Uncooperative patient.
- Obstructive nephropathy with hydronephrosis (9).

## Preparation for renal biopsy

To prevent bleeding complications, a series of checks is conducted prior to performing a kidney biopsy. These checks include ensuring adequate blood pressure control in hypertensive patients, with blood pressure controlled below 140/90 mmHg. Blood tests (CBC and coagulation profile) are also conducted to assess the patient’s hemoglobin level, which should be more than 8 g/dl, platelet count >100,000/µL, and INR <1.4. Urinalysis is performed to rule out any infections, and an ultrasound of the kidneys is conducted to assess their position and size. Additionally, the patient’s medication regimen is reviewed to determine if the patient is taking any blood thinners, such as aspirin, clopidogrel, warfarin, rivaroxaban, dabigatran, or heparin. If so, these medications should be discontinued seven days prior to the procedure (5).

Native kidney biopsy should be performed in a setting with facilities for prompt clinical reassessment, imaging, blood-product support, and timely access to interventional radiology. In cases of persistent or uncontrolled bleeding, angiographic embolization may be required; therefore, an established pathway for urgent interventional management should be available.

## Procedure technique

The patient should be fully informed about the procedure’s indication, technique, and potential problems upon arrival at the interventional radiology unit. The indication, technique, and potential complications should be clearly explained to the patient before the procedure, how the procedure is performed, and how long it takes. All complications should be mentioned to the patient, especially the risk of bleeding, which is increased in patients with vasculitic conditions such as SLE, and how this could be managed should also be well explained to the patient. Patients should also be informed that clinically significant bleeding may require blood transfusion, bladder catheterization and irrigation in cases of gross hematuria with clot retention, angiographic embolization for persistent hemorrhage, or, rarely, emergency surgical intervention. Subsequently, a complete formal consent is obtained from the patient.

**Figure 1:**
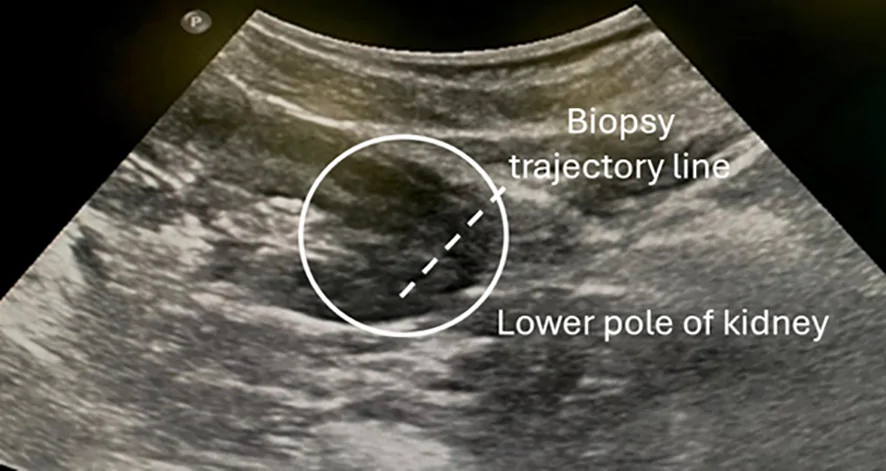
A longitudinal ultrasound scan is done before the biopsy procedure, showing the lower part of the kidney “target for biopsy”, and planning the direction of the biopsy needle pre-procedure.

**Figure 2:**
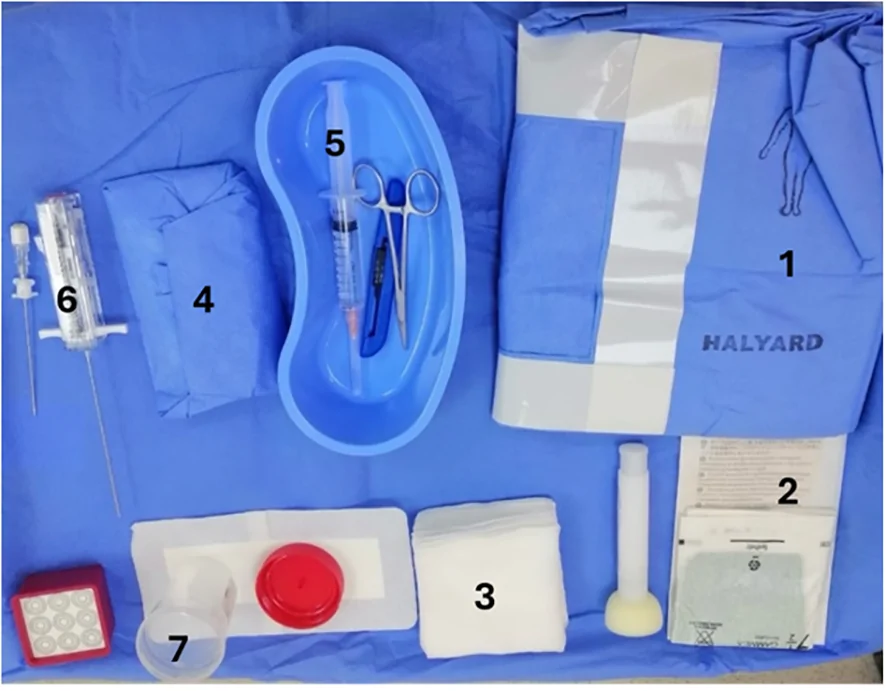
Materials needed for the procedure. 1. Sterile field, 2. sterile gloves, 3. sterile gauze, 4. sterile ultrasound probe cover, 5. local anesthesia in a 10 ml syringe, 6. 16 or 18 gauge x 16 cm biopsy needle, and 7. container.

Placing a pillow beneath the patient’s belly will help them lie prone on a bed. Prior to the surgery, an ultrasound scan should be conducted to determine the optimal site for needle insertion and the proper path for the biopsy needle to follow as it is advanced (**Figure 1**). Subsequently, the right or left back and flank areas should be completely sterilized. A sterile perforated sheet is placed over this area (**Figure 2**). The ultrasound 5–7 MHz curved probe is prepared by applying gel on it, then a sterile probe cover should be applied. The operator must maintain full sterile technique (sterile gown and gloves). The procedure starts with the a repeat quick scan is performed to reconfirm the target site. Local anesthesia is administered under ultrasound guidance until the capsule of the kidney with 22 Gauge x 9.9 cm spinal needle. Then, a 16-, or 18-gauge (outer diameter of 1.65, and 1.27 mm respectively) disposable, automatic spring-loaded true cut core biopsy needle is used (Length of the sample should be at least 20 mm). Biopsies using 16-gauge needles resulted in a mean of 14.5 glomeruli. In contrast, biopsies with 18-gauge needles resulted in a mean of 12 glomeruli (p < 0.05) (10). During ultrasound-guided renal biopsy, the lower-pole renal cortex should be targeted, while unnecessary extension of the needle trajectory into the medulla should be avoided. In a retrospective study of 242 ultrasound-guided renal biopsies, medulla-avoiding biopsies yielded more glomeruli per core and fewer inadequate specimens than medulla-targeting biopsies, with a markedly lower rate of bleeding detected on imaging (12% versus 46%, p<0.001). Therefore, the intended biopsy trajectory and tissue requirements should be discussed before the procedure among the operator, treating nephrologist, and pathologist, particularly when medullary tissue may be required for a specific diagnostic question (11).

**Figure 3:**
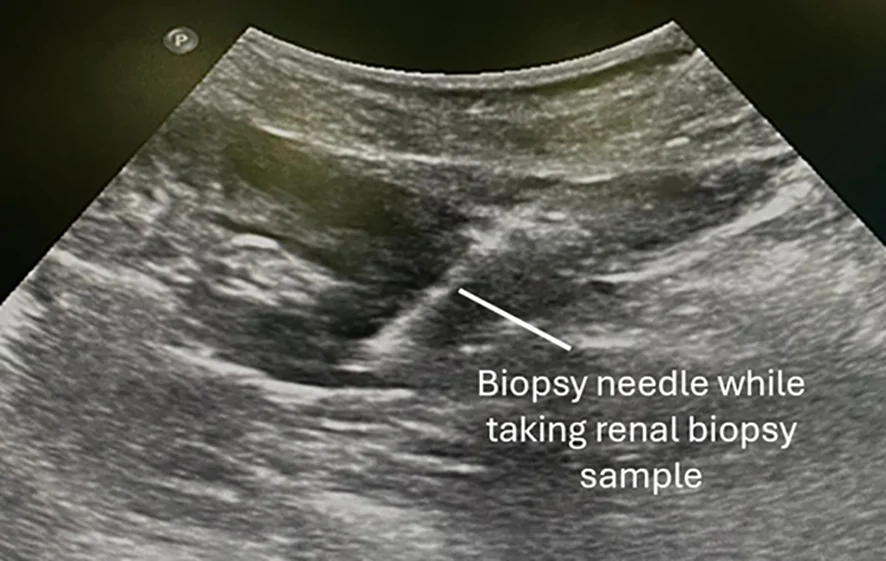
Ultrasound image while obtaining the renal biopsy showing the biopsy needle (Hyperechoic line perpendicular to the long axis of the kidney) fired in the renal cortex.

**Figure 4:**
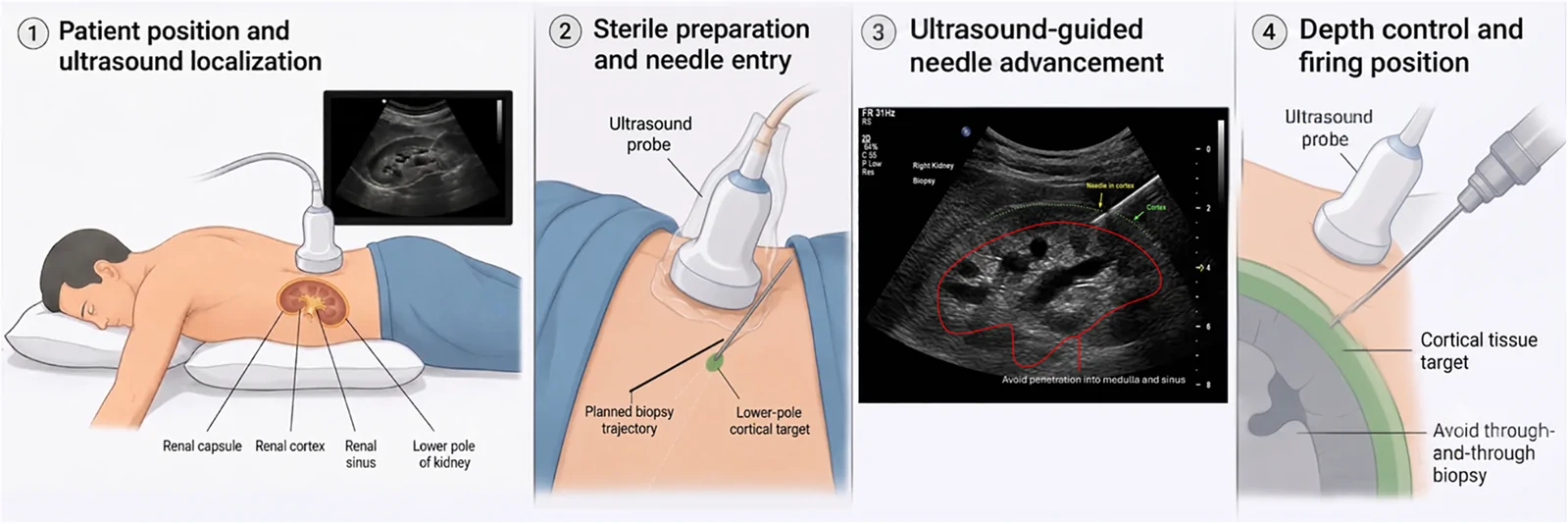
Stepwise protocol for ultrasound-guided native kidney biopsy to maximize safety and yield. This figure was generated using generative Artificial Intelligence (AI) technology.

Under ultrasound guidance, the biopsy needle is advanced carefully with continuous visualization of its tip, while avoiding intrarenal blood vessels. The needle tip should be gently seated in the renal capsule before firing to provide anchorage and minimize deflection, particularly in firm or diseased kidneys. The trajectory should be directed toward the superficial renal cortex and should avoid excessive penetration into the medulla. Then, the biopsy needle is fired (**Figure 3**) and the core is obtained in the pathology containers. The number of biopsy cores should be determined by the clinical indication and the tissue required for histopathological assessment rather than by a fixed procedural target. Before biopsy, the operator should confirm with the treating nephrologist and pathologist whether tissue is required for light microscopy, immunofluorescence, and electron microscopy, as this affects specimen allocation and the number of cores needed (12). The aim is to obtain adequate cortical tissue containing diagnostically useful glomeruli for the required investigations, while avoiding unnecessary additional passes (**Figure 4**). In renal transplant biopsy, Boban and Tiefenthaler reported that two evaluable tissue cores achieved Banff 97 adequacy in 93.1% of biopsy events; however, these findings relate specifically to allograft biopsy and should not be directly extrapolated to native kidneys (13). Finally, an ultrasound scan is performed to check for immediate complications (14).

To promote biopsy site hemostasis, manual pressure is performed at the end of the process. Physicians should explicitly state the following in their postoperative orders: complete supine bed rest for a minimum of four hours, monitoring vital signs every half an hour for the first two hours, and then every hour for the subsequent six hours. Furthermore, it is important to monitor the biopsy site for the development of hematoma or bleeding.

Although the patient should be admitted for at least 24 hours and the procedure is performed as an inpatient intervention, a study conducted by Ivan D Maya et al. showed that outpatient observation for 8 hours post-biopsy without hospitalization is safe (15).

## Other techniques of kidney biopsy

Despite its disadvantages, the transjugular approach allows biopsies of multiple organs during the same procedure and can be recommended when the percutaneous approach fails, and in patients with a severely deranged coagulation profile or bleeding tendency, as it allows selective embolization if bleeding supervened. Transurethral biopsy may be considered in cystoscopy patients who do not wish to undergo percutaneous kidney biopsy separately or when upper urinary tract involvement is suspected (2).

## Complications

Although renal biopsy is considered minimally invasive, complications can still arise. These events are categorized as minor or major, depending on their severity, requiring different management approaches. Hematuria, mild perirenal hematomas, arteriovenous fistulae, and pain are examples of minor issues that are usually resolved conservatively (16). Major complications include significant hematuria with urinary tract obstruction, extensive perirenal hematomas causing incapacitating pain, and bleeding associated with hemodynamic instability. Management is usually required in such situations (17).

Reported complication rates should be interpreted separately for native and transplant kidney biopsies, as patient characteristics, definitions of clinically significant bleeding, and post-biopsy surveillance protocols differ among studies. In a meta-analysis of 118,064 native kidney biopsies, hematoma, macroscopic hematuria, blood transfusion, and interventions to control bleeding were reported in 11%, 3.5%, 1.6%, and 0.3% of biopsies, respectively (18). In contrast, a systematic review of 40,082 renal allograft biopsies reported pooled rates of 3.18% for gross hematuria, 0.31% for transfusion-requiring bleeding, and 0.89% for major complications. These estimates should not be considered universal, as complication frequencies vary according to patient selection, clinical setting, complication definitions, and monitoring practices (19).

According to González-Michaca et al., 2.4% of patients experienced major complications, and 8.65% experienced minor complications, with perirenal hematomas being the most common (20). Bleeding is the most common complication and affects almost all patients (to different degrees). It usually occurs in the first 12 to 24 hours following the procedure (21).

## Post-biopsy monitoring and management of bleeding complications

Following renal biopsy, patients should be monitored clinically for flank pain, hematuria, hemodynamic instability, and features suggestive of ongoing bleeding. A small, stable perirenal hematoma detected on immediate ultrasound in an asymptomatic patient may be managed conservatively with continued observation (22). Worsening pain, gross hematuria, a decrease in hemoglobin concentration, hemodynamic instability, or enlargement of a hematoma should prompt further clinical, laboratory, and imaging assessment. Management of significant bleeding may include fluid resuscitation and blood transfusion when clinically indicated. Bladder catheterization and irrigation may be required in patients with gross hematuria complicated by clot retention. Persistent or clinically significant hemorrhage should be managed in collaboration with interventional radiology, with selective angiographic embolization considered when active bleeding is identified. Surgical intervention is reserved for rare cases in which bleeding cannot be controlled by less invasive measures (23).

## Risk factors for increased rate of complications

A prospective analysis study conducted by Shihao Xu et al. of 218 patients found that patients with immediate post-biopsy active bleeding, a body mass index ≥28 kg/m^2^, and female patients were more likely to suffer perirenal hemorrhage after ultrasound-guided PRB (24).

Systolic hypertension, diastolic hypertension, and increased mean arterial pressure (MAP) were identified as significant risk factors for bleeding problems in a retrospective study of 645 native renal biopsies performed by Shidham et al. (6).

Poor renal function has been associated with a higher risk of complications in many studies (25–27). Although they appeared sicker, transplanted kidneys had fewer serious complications than native kidneys post-biopsy (28).

## Conclusion

Ultrasound-guided renal biopsy is an essential diagnostic procedure for renal parenchymal diseases and plays a crucial role in guiding patient management. The technique has a high technical success rate, while major complications remain uncommon. Successful and safe performance of the procedure depends on appropriate patient selection, careful pre-procedural preparation, operator expertise, and structured post-biopsy monitoring. A clear understanding of the indications, contraindications, procedural steps, and potential complications is essential for operators performing this procedure.
